# Genetic analysis of grain yield and related traits of extra-early orange maize inbred lines and their hybrids under drought and rain-fed conditions

**DOI:** 10.3389/fpls.2024.1463924

**Published:** 2024-11-29

**Authors:** Tégawendé Odette Bonkoungou, Baffour Badu-Apraku, Victor Olawale Adetimirin, Kiswendsida Romaric Nanema, Idris Ishola Adejumobi

**Affiliations:** ^1^ Pan African University Life and Earth Sciences Institute (including Health and Agriculture), University of Ibadan, Ibadan, Oyo State, Nigeria; ^2^ International Institute of Tropical Agriculture (IITA), Ibadan, Nigeria; ^3^ Department of Crop and Horticultural Sciences, University of Ibadan, Ibadan, Oyo State, Nigeria; ^4^ Biosciences Laboratory, Joseph KI-Zerbo University, Ouagadougou, Burkina Faso

**Keywords:** drought stress, general combining ability, genetic distance, heterosis, specific combining ability

## Abstract

**Background:**

Orange maize genotypes are sources of provitamin A (PVA) carotenoids, which are precursors of vitamin A. PVA deficiency and drought constitute major challenges causing increasing food and nutritional insecurity in sub-Saharan Africa (SSA). Breeding of drought-tolerant provitamin A hybrid maize can mitigate these challenges. This study was undertaken to determine the combining ability of newly developed extra-early orange inbreds for grain yield and related traits under managed drought stress and rain-fed conditions, determine the mode of gene action conditioning the inheritance of the traits, and classify the inbreds into heterotic groups.

**Methodology:**

One hundred and ninety-six extra-early orange hybrids comprising 180 testcrosses, 10 single crosses, and 6 commercial checks were evaluated under managed drought and rain-fed conditions at Ikenne. In addition, 41 inbreds comprising 36 orange lines and 5 PVA testers involved in the hybrid development were assessed under drought and rain-fed conditions.

**Results:**

The means square for general combining ability (GCA) and specific combining ability (SCA) were significant for grain yield and most other traits under both growing conditions. The contributions of GCA to performance were larger than SCA in each growing condition. Broad-sense and narrow-sense heritability estimates for grain yield were 66% and 37% under managed drought and 88% and 32% under rain-fed conditions, respectively. Mid-parent heterosis and better-parent heterosis for grain yield were 338% and 247% under managed drought, while 173% and 137% under rain-fed conditions. Significant positive correlations existed among grain yield of hybrids, heterosis, and specific combining ability under managed drought. The 41 inbred lines were classified into three heterotic groups under both growing conditions. Sixteen testcross hybrids out-yielded the best commercial check under managed drought.

**Conclusion:**

The testcross hybrids have great potential for commercialization to address the problem of drought and PVA deficiency in SSA. Inbred TZEEIOR 510 showed desirable GCA effects for grain yield and 04 other traits under drought.

## Introduction

Maize is an economically important cereal crop worldwide due to its multiple uses as human food, animal feed, and raw material for a wide range of manufacturing products (Prasanna et al., 2020). It is a major life sustenance crop for about 1.2 billion people in Latin America and SSA (International Institute of Tropical Agriculture, 2010; Nuss and Tanumihardjo, 2010). Numerous households in SSA frequently use maize as the main weaning diet that excludes proteins derived from livestock food in resource-limited rural communities (Manjeru et al., 2019). The nutritional importance of the crop particularly varieties derived from yellow and orange endosperm germplasm, is due to the presence of nutrients in their kernels (Manjeru et al., 2019).

White maize kernel, which many consumers use for the preparation of many dishes, lacks carotenoids (Nuss and Tanumihardjo, 2010; Siwela et al., 2020; Msungu et al., 2022). Consequently, consumers of white kernel maize are very prone to health challenges relating to vitamin A deficiency (VAD) (Ekpa et al., 2018). During the past two decades, tremendous efforts have been made to improve the nutritional status of several crops consumed in SSA including maize, but the region still has the largest number of malnourished people in the world. For instance, in West and Central Africa (WCA), a large proportion of the population has limited access to nutritionally balanced food to support a healthy life (Badu-Apraku and Fakorede, 2017). Maternal and childhood malnutrition results in underweight individuals, causing millions of deaths in the sub-region. The World Health Report of 2002 ranked malnutrition first among the top globally preventable health risks, with the dreaded HIV/AIDS ranking fourth, an indication that greater attention should be focused on improving nutrition to minimize the effect of preventable diseases. The most promising strategy for addressing the health issues resulting from VAD is biofortification. Parental inbred lines with improved levels of provitamin A (PVA) can facilitate the development of hybrid maize varieties with improved levels of PVA contents (beta carotenoid), which is a major precursor of retinol (Saltzman et al., 2013). Increased PVA content is associated with the orange color of the maize endosperm (Beta et al., 2014; Hwang et al., 2016) and such maize varieties are referred to as orange maize. Previous studies (Lieshout and West, 2005; Nuss et al., 2012; Bouis and Saltzman, 2017) have demonstrated that orange maize cultivars have improved levels of PVA that address the health issues related to VAD.

In addition to VAD, the yields of many maize varieties are substantially lower on farmers’ fields compared to the yield observed under optimum growing conditions (Cairns et al., 2013; Masasi and Ng’ombe, 2019). This is because of the exclusive dependence of many farmers on rainfall conditions (Kelvin and Ng’ombe, 2016). Drought stress affects maize at all developmental stages. Flowering and grain-filling stages are the most susceptible (Sheoran et al., 2022). Yield losses between 40-90% have been reported when the drought stress coincides with flowering and grain-filling (Chen et al., 2010; Széles et al., 2023). Over seventy PVA varieties combining hybrids and open-pollinated varieties have been released in Africa (Harvestplus, 2024). However, drought-tolerant extra-early PVA hybrids are limited. To address vitamin A deficiency (VAD) and food insecurity in SSA, the development and commercialization of drought-tolerant maize with high provitamin A (PVA) content is urgently needed. Significant progress has been made in increasing PVA in maize through conventional breeding. As highlighted by Badu-Apraku and Fakorede (2017), identifying adapted orange maize inbred lines from diverse genetic backgrounds with varying carotenoid concentrations is crucial for developing superior PVA hybrids and establishing a successful PVA breeding program. Therefore, generating PVA or orange drought-tolerant extra-early maize would contribute successfully to combat the combined challenges of drought stress and low nutritional quality in marginal rainfall areas in SSA.

Understanding inter-trait relationships is crucial for breeders to select the most effective traits for improving grain yield under diverse environmental conditions (Talabi et al., 2017). Researchers routinely investigate how grain yield and secondary traits interact, especially when working with new genetic materials, to ensure that existing relationships are not affected by genetic changes or climate change. Previous studies have identified several secondary traits associated with grain yield improvement. Bänziger et al. (2000) found that number of ears per plant (EPP), anthesis-silking interval (ASI), and stay-green characteristic (STGC) are reliable predictors of grain yield under drought and low N conditions. Badu-Apraku et al. (2011) also identified EPP, ASI, plant aspect (PASP), and ear aspect (EASP) as important secondary traits for yield improvement under both drought and low N stresses. Additionally, they found that days to silking (DS), days to anthesis (DA), plant height (PHT), and STGC can be used as indirect selection criteria for grain yield under low-N environments.

Since 2007, several inbred lines with varying levels of PVA and reactions to drought are being developed in the International Institute of Tropical Agriculture (IITA) maize improvement program. The potential utilization of these inbreds in hybrid combinations is continuously determined by their classification into appropriate heterotic groups (Badu-Apraku et al., 2020; Aslam and Zafar, 2017; Meena et al., 2017). This enables a better understanding of the genetic relationships among the inbreds for effective utilization in the development of hybrids maize, synthetic varieties, and heterotic populations. For effective hybrid maize development, planning and efficient gene-deployment schemes in the breeding program, knowledge of the mode of gene action controlling the inheritance of grain yield and other secondary traits is important (Amegbor et al., 2020; Temesgen, 2021). In SSA where only few PVA hybrids in the extra-early maturity group have been developed and released for commercialization, this information is crucial for the development of more extra-early maturity group PVA drought-tolerant (DT) hybrids for commercialization. The studies reported here were conducted to determine (i) the combining ability for grain yield and other agronomic traits, (ii) the mode of gene action for these traits under different environmental conditions, (iii) the inter-trait correlations, (iv) the appropriate heterotic groups for the inbred lines, and (v) the hybrids with outstanding yield potential above commercially grown varieties.

## Materials and methods

### Experimental location

The drought experiments were conducted during the dry seasons between December 2021 and March 2022, and November 2022 and February 2023 at the IITA experimental sub-station at Ikenne, Ogun state, Southwestern Nigeria. The experiments conducted during this period were entirely dependent on irrigation. Ikenne (6053’N, 30 42 E, 60 m altitude) is in the lowland humid forest zone. The experimental station comprises strongly leached, highly weathered, and well-drained loamy sand eutric nitisols (FAO/UNESCO, 1994) with flat and uniform terrain. The rainfall in the southwestern part of Nigeria where Ikenne is located, is relatively high, with a bimodal pattern (average annual total of 1732 m) spanning from April to October. The daily rainfall data from December 2021 to March 2022 and November 2022 to February 2023, averaged 0.9 and 1.2 mm, respectively (**Supplementary Figure S1**). The daily minimum temperature varied from 14.3 to 25°C with an average of 21.9°C from December 2021 to March 2022 and from 16.6 to 24.2°C with an average of 22°C from November 2022 to February 2023. The daily maximum temperature ranged from 26.9 to 32.9°C with an average of 30.4°C from December 2021 to March 2022 and from 27.6 to 33.9°C with an average of 30.6°C from November 2022 and February 2023 (**Supplementary Figure S2**). Given that Ikenne is practically rain-free during the dry season, the withdrawal of irrigation water at specific growth stages of the maize crop allows the management of the drought intensity. The land use is characterized by intense small-scale farming. The rain-fed experiments were conducted during the rainy season from June to September 2022 and 2023 at the same location.

### Genetic materials

To develop drought-tolerant, extra-early maturing maize cultivars with high provitamin A content for farmers in SSA, the *Striga*-resistant cultivar 2004TZEE-Y STR C_4_ was crossed with the high-provitamin A source SYN-Y-STR-34–1-1–1-1–2-1-B-B-B-B-B/NC354/SYN-Y-STR-34–1-1–1 in 2007. The F_1_ generation was backcrossed to the extra-early cultivar and selection was made based on deep orange-colored kernels in the BC_1_F_1_ generation. Through selfing, selection, and recombination at F_3_, the extra-early PVA cultivar 2009 TZEE-OR1 STR was developed in 2011. Several inbred lines were extracted from this population by 2014 with deep orange color. To assess drought tolerance, these inbred lines alongside inbreds from other source populations were evaluated under induced drought conditions during the 2014-2015 dry season in Ikenne, Nigeria and the promising lines from the evaluation were advanced to S_8_. Of the S_8_ inbred lines, we selected a panel of 210 inbred lines from where 41 inbred lines were further selected for this study. The 41 inbred lines comprised 16, 11, and 15 inbreds from the source populations 2009 TZEE-OR1 STR, TZdEEI 12 × TZEEI 95, and TZdEEI 7 × TZdEEI 12, respectively. Of the selected 41 inbred lines, five were used as standard testers in the extra-early section of the IITA maize improvement program leaving 36 as lines (**Supplementary Table S1**). The 36 lines were crossed with the 5 testers to generate 180 testcross hybrids. Six commercial varieties (five single cross and one topcross hybrids) were added to the testcross hybrids to make 186 hybrids. In an attempt to ensure efficiency, balance, and simplicity in trial evaluation by approaching a square design in alpha lattice, we crossed the testers in half-diallel to develop 10 hybrids, which were added to the 186 hybrids to make 196 hybrids.

### Field evaluation

The hybrids and inbred lines trials were evaluated for agronomic performance at Ikenne (7°52′ N, 30°44′ E, 61 m a.s.l., 1200 mm mean annual precipitation) in the forest ecological zone of Nigeria. The trials were conducted under managed drought in the dry seasons from 2021-2022 and 2022-2023 and under rain-fed conditions in 2022 and 2023 major season. Under the managed drought conditions, water was provided through sprinkler irrigation system which applied 17 mm water per week for 25 days after planting (DAP). Thereafter, the plants were allowed to depend on the available soil moisture till they attained physiological maturity. The rain-fed conditions depended on natural rain. The hybrid trials were laid out using a 14 × 14 alpha lattice design in two replicates and the inbred lines trials (210) were laid out using 14 × 15 alpha lattice design in two replicates under each growing condition. The plot size was single rows of 3 m length with inter and intra-row spacing of 0.75 and 0.40 m, respectively. During planting, three seeds were sown per hill and thinned to two seedlings per hill about 2 weeks after emergence to give a plant population density of 66,666 plants/ha. The maize nutrients nitrogen (N), phosphorus (P), and potassium (K) was applied at the rate of 120:60:60 kg/ha of N, P, and K, respectively. To achieve this rate, a compound fertilizer (NPK 15:15:15) was first applied at 12g per hill to obtain an application rate of 60 kg/ha rate of N, P, and K, respectively at planting for managed drought trials while at 14 days after planting (DAP) for the rain-fed trials. Additional 4 g of Urea (46:0:0) fertilizer was applied per hill to obtain an application rate of approximately 60 kg/ha of N as a top-dress at three weeks after planting (WAP) for the managed drought trials and at 5 WAP for the rain-fed trials. In all the trials, after pre-emergence weed control using a combination of contact (gramoxone 5 liter/ha (paraquat)) and systemic (primextra 5 liter/ha (glyphosate)) herbicides, hand weeding was done at 3 WAP and subsequent weed control was done using weedicides (gramoxone 5 liter/ha and primextra 5 liter/ha) to keep the fields weed-free throughout the experiment period.

### Data collection

Data were collected on grain yield (GY), days to 50% pollen shed (DP), days to 50% silking (DS), anthesis-silking interval (ASI), plant height (PHT), ear height (EHT), ear per plant (EPP), plant aspect (PASP), ear aspect (EASP), stay green characteristic (STGC) and husk cover (HCV) as described by Bonkoungou et al. (2024). Plants aspect (PASP) was rated on a scale of 1–9, where 1 = excellent and 9 = poor. Ear aspect (EASP) was recorded on a scale of 1–9, where 1 = clean, uniform, large, and well-filled ears and 9 = ears with undesirable features, such as diseases, small ears, ears rot, and ears with poorly filled grains. Stay green characteristic (STGC) or leaf death score (LD) was determined under drought-stress conditions at 70 days after planting (DAP) on a scale of 1 to 9, where 1 = almost all leaves green and 9 = virtually all leaves dead. Husk cover (HCV) was rated on a scale of 1 to 9, where 1 = husks tightly arranged and extended beyond the ear tip and 9 = ear tips exposed.

### Data analysis

Before subjecting the data obtained from the evaluation to genetic analysis in AGD-R software (version 5.1), the 10 hybrids from the half-diallel were removed leaving the testcrosses and the commercial varieties. A combined analysis of variance (pooled ANOVA from two growing conditions and two seasons) and growing condition specific, ANOVA were performed following the line-by-tester model below.


$$Y_{ijkl}=\mu +G_{i}+T_{j}+G_{jk}+G\times E_{\left(ik\right)}+E_{ijkl}$$

*Where; Y_ijkl_ = phenotype of the individual from inbred line i and tester j in growing condition k and season l; μ = overall mean; G_i_ = general combining ability (GCA) effect of inbred line i; T_j_ = general combining ability (GCA) effect of tester j; G_jk_ = specific combining ability (SCA) effect of the cross between inbred line i and tester j in growing condition k; GxE_(ik)_ = genotype by environment interaction effect for inbred line i in growing condition k, E_ijkl_ = random error term.*

To analyze the source of variation in the testcrosses, we partitioned the variance into three components: the general combining ability of inbred lines (GCA Line), the general combining ability of testers (GCA Tester), and the specific combining ability (SCA Line x Tester). We calculated the percentage contributions of GCA (GCA Line + GCA Tester) and SCA variances to the total genetic variance based on the mean square of the SCA+GCA components. A ratio closer to unity indicates greater predictability based on general combining ability alone (Baker, 1978). We also estimated broad-sense heritability (H^2^) and narrow-sense heritability (h^2^) for each trait under each growing condition using the same software and the following formula


$$H^{2}=\frac{\sigma_{A}^{2}+\sigma_{D}^{2}}{\sigma_{A}^{2}+\sigma_{D}^{2}+\sigma_{E}^{2}}$$

Where *H^2^ = broad-sense heritability; σ^2A^ =additive variance; and σ^2D^ = dominance variance; σ^2E^=environmental variance*


$$h^{2}=\frac{\sigma_{A}^{2}}{\sigma_{A}^{2}+\sigma_{D}^{2}+\sigma_{E}^{2}}$$

Where *h^2^ = narrow-sense heritability; σ^2A^ =additive variance; and σ^2D^ = dominance variance; σ^2E^=environmental variance*

After the genetic analysis, we proceeded with assigning the inbred lines into distinct heterotic groups under the contrasting growing conditions using the Heterotic Grouping procedure based on the General Combining Ability of Multiple Traits (HGCAMT) proposed by Badu-Apraku et al. (2013). We use R studio version 4.3.1 (R Core Team, 2023) and its associated packages to handle the rest of the analysis. To facilitate heterotic grouping, we standardized the GCA effects of traits with significant genotype mean squares under each growing condition using Z-score transformation using the tidyverse package (Wickham et al., 2019) implemented in R. This process ensured comparability across traits with different scales. From the standardized GCA values, we extracted Euclidean distance and subjected it to Ward.D2 clustering method using the hclust function in R. The optimum number of clusters was identified using the package NbClust (Malika et al., 2014) in R, and the final dendrogram revealing the pattern and cluster members was plotted using the dendextend package (Galili, 2015) in R. Following the dendrogram construction showing the heterotic pattern of the 41 inbreds for both managed drought and rain-fed conditions, a comparison of the two dendrograms (managed drought and rain-fed) was made using the tanglegram function in the dendextend package (Galili, 2015) in R to visualize the relationships.

Following heterotic grouping, we extracted the Best Linear Unbiased Estimates (BLUEs) from the ANOVA model under each growing condition for the hybrids and inbred lines. The BLUEs were used to estimate heterosis (mid-parent, better parent, and economic heterosis) for the traits where significant genotype mean squares were observed as shown in the equations below:


$$Mid-parent\;performance=MP=\frac{P1+P2}{2}$$

$$Mid-parent\;heterosis\;MPH(\%)=\frac{F1-MP}{MP}*100$$

$$Better-parent\;heterosis=BPH\left(\%\right)=\frac{F1-BP}{BP}*100$$

*Where MP = Mid-parent; P_1_ = parent 1, P_2_ = parent 2, BPH = best parent heterosis; BP=better- parent; F_1_= testcross performance*

The economic heterosis for grain yield under managed drought conditions was calculated as follow:


$$EH\left(\%\right)=\frac{F1-BCC}{BCC}*100\;$$

*Where, EH= economic heterosis; F_1_= grain yield of hybrid, BCC= grain yield of best commercial variety*

The relationship between the performance of the testcrosses (F_1_) and their MP was computed for all the traits with significant mean square using the Metan package (Olivoto and Lúcio, 2020) in R for each growing condition. Additionally, the correlation between the grain yield performance of the test-crosses (F_1_) and MP, BP, MPH, BPH, and SCA effects were estimated.

## Results

### Analysis of variance and combining ability for grain yield and related traits of extra-early orange hybrids under drought and rain-fed conditions

The mean square from the analysis of variance of the pooled data revealed significant genotype and environment effects (p<0.001) for all the measured traits. The genotype × environment (GEI) mean square was significant (p<0.001) for GY, ASI, PHT, PASP, EASP, HCV, and EPP (**Supplementary Table S2**). Under managed drought conditions, mean squares for genotypes (G) were significant (p < 0.001) for all the traits. Environment mean square was significant (p< 0.001) for the measured traits except DS, STGC, and EHT (**Table 1**). The mean square of GEI was significant (p<0.001) for ASI, PHT, HCV and EPP. When the genotypic mean square was portioned into GCA Line, GCA Tester and SCA Line × Tester, significant (p< 0.05) GCA Line, GCA Tester and SCA Line × Tester were observed for all of the traits except SCA Line × Tester for ASI and EASP. Mean square of GCA Line × environment interaction (GCA Line × E) was significant (p< 0.05) for ASI, PHT, EASP, and HCV while GCA Tester × environment interaction (GCA Tester × E) was significant (p< 0.05) for GY, DP, PASP, EASP, HCV, EPP, PHT and EHT. The mean square of SCA Line × Tester × environment was significant (p< 0.05) for only EASP (**Table 1**).

**Table 1:** Mean squares for grain yield and other agronomic traits of extra-early orange maize hybrids evaluated under managed drought stress and rain-fed conditions at Ikenne in two seasons.

Source	DF	GY		DP		DS		ASI		PHT		
		Drought	rain-fed	Drought	Rain-fed	Drought	Rain-fed	Drought	Rain-fed	Drought	Rain-fed	
E	1	16953301.13***	114277177.06***	38.09***	44.3***	9.76^ns^	96.61***	422.76***	71.57***	8597.12***	7.77^ns^	
REP(E)	2	9174207.52***	2980943.84***	4.83^ns^	1.37^ns^	29.08*	1.53^ns^	18.43***	0.51^ns^	2328.32***	44.02^ns^	
GENOTYPES	179	1530491.13***	4209073.7***	8.58***	4.25***	18.99***	8.37***	3.073***	0.74***	510.16***	413.72***	
LINE (GCA Line)	35	3246398.69***	7090612.65***	14.78***	7.53***	40.39***	19.62***	6.66***	0.74***	1119.07***	681.65***	
TESTER(GCA Tester)	4	3662333.70***	33845679.66***	115.01***	35.27***	169.34***	44.62***	6.03*	5.03***	3846.09***	7953.42***	
LINE × TESTER(SCA Line × Tester)	140	1042447.91*	2642059.35***	3.99***	2.54***	9.35***	4.53***	2.09^ns^	0.62***	262.85*	131.32***	
GENOTYPES × E	179	920121.23^ns^	1197812.93^ns^	2.52^ns^	1.31*	5.55^ns^	1.83^ns^	2.17*	0.43^ns^	243.14*	69.82^ns^	
GCA LINE × E	35	1073615.32^ns^	1658989.67*	2.73^ns^	1.39^ns^	6.46^ns^	1.87^ns^	4.08***	0.62*	369.94***	93.94^ns^	
GCA TESTER × E	4	2057867.95*	2948088.14*	6.23*	2.74*	12.77^ns^	2.5^ns^	1.45^ns^	0.43^ns^	1011.91***	116.77^ns^	
Line × Tester × E	140	849240.66^ns^	1032510.89^ns^	2.37^ns^	1.25^ns^	5.12^ns^	1.8^ns^	1.71^ns^	0.38^ns^	189.47^ns^	62.45^ns^	
Residuals	306	823214.9	1095273.8	2.45	1.03	5.66	1.74	1.66	0.39	192.69	71.65	
Source	DF	EHT		PASP		EASP		HCV		EPP		STGC
		Drought	Rain-fed	Drought	Rain-fed	Drought	Rain-fed	Drought	Rain-fed	Drought	Rain-fed	Drought
E	1	13.05 ^ns^	127.85 ^ns^	43.04***	23.76***	36***	9.34***	71.41***	24.65***	2.95***	1***	1.30^ns^
REP(E)	2	696.65***	109.36^ns^	5.60***	0.04^ns^	15.25***	2.62*	0.44^ns^	5.64***	0.40***	0^ns^	7.76***
GENOTYPES	179	212.51***	198.5***	1.63***	2.07***	1.66***	0.88^ns^	3.90***	7.06***	0.06***	0.02***	2.02***
LINE (GCA Line)	35	389.46***	321.46***	2.18***	2***	3.37***	0.7^ns^	5.96***	6.71***	0.11***	0.02***	2.30***
TESTER (GCA Tester)	4	2107.35***	2763.33***	9.62***	31.4***	5.04***	4.05***	83.22***	204.02***	0.29***	0.12***	20.98***
LINE × TESTER (SCA Line × Tester)	140	114.16***	94.49***	1.27*	1.25***	1.14^ns^	0.83^ns^	1.12***	1.52***	0.03*	0.02***	1.41***
GENOTYPES × E	179	72.85^ns^	55.59 ^ns^	1.03^ns^	0.65^ns^	1.09^ns^	1*	0.98*	0.79^ns^	0.04*	0.01^ns^	1.03^ns^
GCA LINE × E	35	70.75^ns^	81.39*	1.33^ns^	0.74^ns^	1.47*	1.15*	1.28*	0.97 ^ns^	0.03^ns^	0.01^ns^	0.71^ns^
GCA TESTER × E	4	377.3***	71.12^ns^	2.67*	4.23***	2.74*	4.23***	3.37***	3.19***	0.31***	0.04*	0.67^ns^
LINE × TESTER × E	140	64.68^ns^	48.7^ns^	0.91^ns^	0.53^ns^	0.95*	0.87^ns^	0.83^ns^	0.68	0.03^ns^	0.01^ns^	1.13^ns^
Residuals	306	69.55	51.07	0.94	0.86	0.98	0.74	0.77	0.81	0.03	0.01	0.98

E, environment; GCA, general combining ability; SCA, specific combining ability; GY, grain yield; ASI, anthesis-silking interval; PHT, plant height; EHT, ear height; PASP, plant aspect; EASP, ear aspect; EPP, ear per plant; HCV, husk cover; DP, days to 50% pollen shed; DS, days to 50% silking; *, significant at 0.05 probability level; **, significant at 0.01 probability level; ***, significant at 0.001 probability level; ns, non-significant.

Under rain-fed condition, significant (p< 0.001) genotype mean square was observed for all the traits except EASP. The environment mean square was significant (p< 0.001) for all the traits except PHT and EHT. The mean square of GEI was significant (P<0.05) for only DP and EASP. The mean squares of GCA Line, GCA Tester and SCA Line × Tester were significant (p<0.001) for all the traits except for EASP where non-significant effect was observed for GCA Line and SCA Line × Tester. The mean square of GCA Line × environment interaction was significant (p< 0.05) for GY, ASI, EHT, and EASP while that of GCA Tester × environment interaction was significant (p< 0.05) for GY, DP, PASP, EASP, HCV, and EPP (**Table 1**).

**Figure 1:**
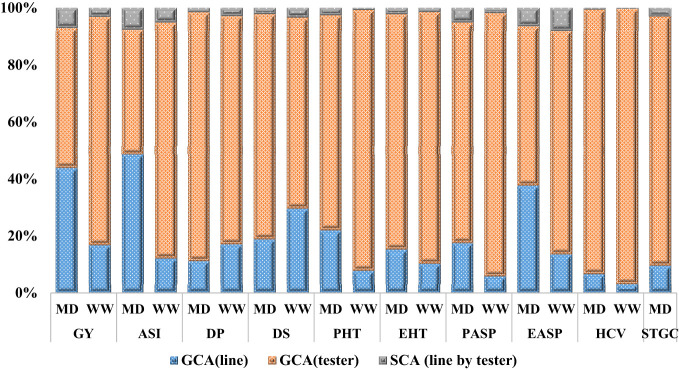
Proportion of additive (GCA) and non-additive (SCA) genetic variances for grain yield and other agronomic traits under managed drought and rain-fed conditions. GCA, general combining ability; SCA, specific combining ability, MD, managed drought; WW, well-watered=rain-fed; GY, grain yield; ASI, anthesis-silking interval; PHT, plant height; EHT, ear height; PASP, plant aspect; EASP, ear aspect; EPP, ear per plant; HCV, husk cover; DP, days to 50% pollen shed; DS, days to 50% silking; STGC, stay green characteristic.

The mode of inheritance for grain yield and other measured traits in the extra-early orange germplasm under each of the growing conditions (managed drought and rain-fed) revealed that the GCA mean square was greater than the SCA mean square for GY and related traits. This suggests that the GCA variance was more important than SCA variance in the expression of the measured traits under each of the growing conditions. The GCA variance accounted for more than 90% of the total genetic variation observed. In addition, the GCA variance of the tester was higher than the GCA variance of the lines and the SCA (line by tester) variance for almost all the traits (**Figure 1**).

### Genetic parameters of agronomic traits under managed drought and rain-fed conditions

The genetic parameter of the measured traits under each of the growing conditions was presented in **Table 2**. Under managed drought conditions, the broad-sense heritability for GY was 66% while a range between 59% (ASI) and 87% (DS) were found for other agronomic traits. Narrow-sense heritability for GY was 37% while a range between 25% (PASP) and 71% (HCV) were found for other agronomic traits. Under rain-fed conditions, broad-sense heritability varied from 26% for EASP to 95% for HCV. Broad-sense heritability for GY was 88%. Narrow-sense heritability varied from 6% for EASP to 67% for PHT, with a value of 32% for GY. Broad-sense heritability estimates were higher under rain-fed conditions than under managed drought stress conditions for all the traits except EASP. Narrow-sense heritability was higher under managed drought than under rain-fed conditions for GY, DP, DS, ASI, EASP, and EPP. However was higher for PHT, EHT, PASP, and HCV under rain-fed compared to managed drought. Among these traits, the largest differences in narrow-sense heritability between managed drought and rain-fed conditions were observed for EASP, ASI, and EPP (**Table 2**). Across traits, broad-sense heritability averaged 0.73 under managed drought and 0.79 under rain-fed conditions. The values for narrow-sense heritability were 0.43 and 0.38, respectively.

Table 2Estimates of variance components and heritability of grain yield and other agronomic traits of extra-early orange maize from data collected in two dry and two rainy seasons in Ikenne, Nigeria.

**Table:** 

Traits	Line variance		Tester variance		Line-by-tester variance		$\sigma_{G}^{2}$		$\sigma_{A}^{2}$	
	Drought	Rain-fed	Drought	Rain-fed	Drought	Rain-fed	Drought	Rain-fed	Drought	Rain-fed
GY	110197.54	222427.66	18193.65	216691.81	54808.25	386696.39	68553999	188360618	274664.01	879353.75
DP	0.54	0.25	0.77	0.23	0.39	0.38	384.18	190.14	2.58	0.96
DS	1.55	0.75	1.11	0.28	0.92	0.7	850.11	374.74	5.41	2.16
ASI	0.23	0.01	0.03	0.03	0.11	0.06	137.51	33.18	0.55	0.07
PHT	42.81	27.52	24.88	54.32	17.54	14.92	22837.97	18514.11	138.87	158.46
EHT	13.76	11.35	13.84	18.53	11.15	10.85	9510.87	8883.2	55.2	58.37
PASP	0.05	0.04	0.06	0.21	0.08	0.1	73.02	92.54	0.2	0.46
EASP	0.11	0	0.03	0.02	0.04	0.02	74.47	39.25	0.29	0.03
HCV	0.24	0.26	0.57	1.41	0.09	0.18	174.68	315.77	1.56	3.11
STGC	0.04		0.14		0.11		90.38		0.34	
EPP	0	0	0	0	0	0	2.49	0.85	0.01	0

σ^2G^, genetic variance; σ^2A^, additive variance; GY, grain yield; ASI, anthesis-silking interval; PHT, plant height; EHT, ear height; PASP, plant aspect; EASP, ear aspect; EPP, ear per plant; HCV, husk cover; DP, days to 50% pollen shed; DS, days to 50% silking.

**Table:** 

Trait	$\sigma_{D}^{2}$		$\sigma_{E}^{2}$		*H^2^*		h^2^	
	Drought	Rain-fed	Drought	Rain-fed	Drought	Rain-fed	Drought	Rain-fed
GY	219233.01	1546785.6	254256.89	325088.02	0.66	0.88	0.37	0.32
DP	1.54	1.51	0.65	0.4	0.86	0.86	0.54	0.33
DS	3.69	2.79	1.42	0.48	0.87	0.91	0.51	0.4
ASI	0.43	0.23	0.67	0.12	0.59	0.72	0.33	0.16
PHT	70.17	59.67	73.4	17.91	0.74	0.92	0.49	0.67
EHT	44.61	43.42	19.04	15.03	0.84	0.87	0.46	0.5
PASP	0.32	0.39	0.28	0.21	0.65	0.8	0.25	0.43
EASP	0.16	0.09	0.3	0.31	0.6	0.26	0.39	0.06
HCV	0.35	0.7	0.3	0.2	0.87	0.95	0.71	0.77
STGC	0.43		0.27		0.74		0.33	
EPP	0.01	0.01	0.01	0	0.65	0.74	0.37	0.17

GY, grain yield; ASI, anthesis-silking interval; PHT, plant height; EHT, ear height; PASP, plant aspect; EASP, ear aspect; EPP, ear per plant; HCV, husk cover; DP, days to 50% pollen shed; DS, days to 50% silking, STGC, stay green characteristic; σ_A_, additive variance; σ^2D^, dominance variance; σ^2E^, environmental variance; σ^2G^, genetic variance.

### Estimates of general combining ability effects of 36 extra-early orange inbreds under drought stress and rain-fed environments

Under managed drought, the GCA effects for grain yield ranged from -670.3 for TZEEIOR 28 to 899.6 for TZEEIOR 510. Inbred TZEEIOR 510 had significant and positive GCA effects for GY and EPP while desirable significant and negative GCA effects were observed for the same inbred for PASP, EASP, and STGC. TZEEIOR 479 had a significant negative GCA effect for DS while TZEEIOR 142 and TZEEIOR 221 had positive significant GCA for ASI. TZEEIOR 28 had significant positive GCA effects for PHT. Significant positive GCA for EHT was observed for TZEEIOR 205 (**Supplementary Table S3**).

Under rain-fed conditions, the GCA effect for GY ranged from -1858 for TZEEIOR 28 to 814.2 for TZEEIOR 546. TZEEIOR 28, TZEEIOR 41, TZEEIOR 45, and TZEEIOR 24 had significant negative GCA effects for GY while TZEEIOR 41 and TZEEIOR 28 had significant positive GCA effects for PASP. A significant positive GCA effect for DP, DS, and PHT was observed for TZEEIOR 142 while TZEEIOR 205 had a significant positive GCA effect for PHT and EHT. TZEEIOR 381 had a significant positive GCA effect for EASP while TZEEIOR 479 had a positive significant GCA effect for ASI (**Supplementary Table S4**).

### Heterotic grouping of parental inbreds evaluated under managed drought and rain-fed conditions using the HGCAMT method

**Figure 2:**
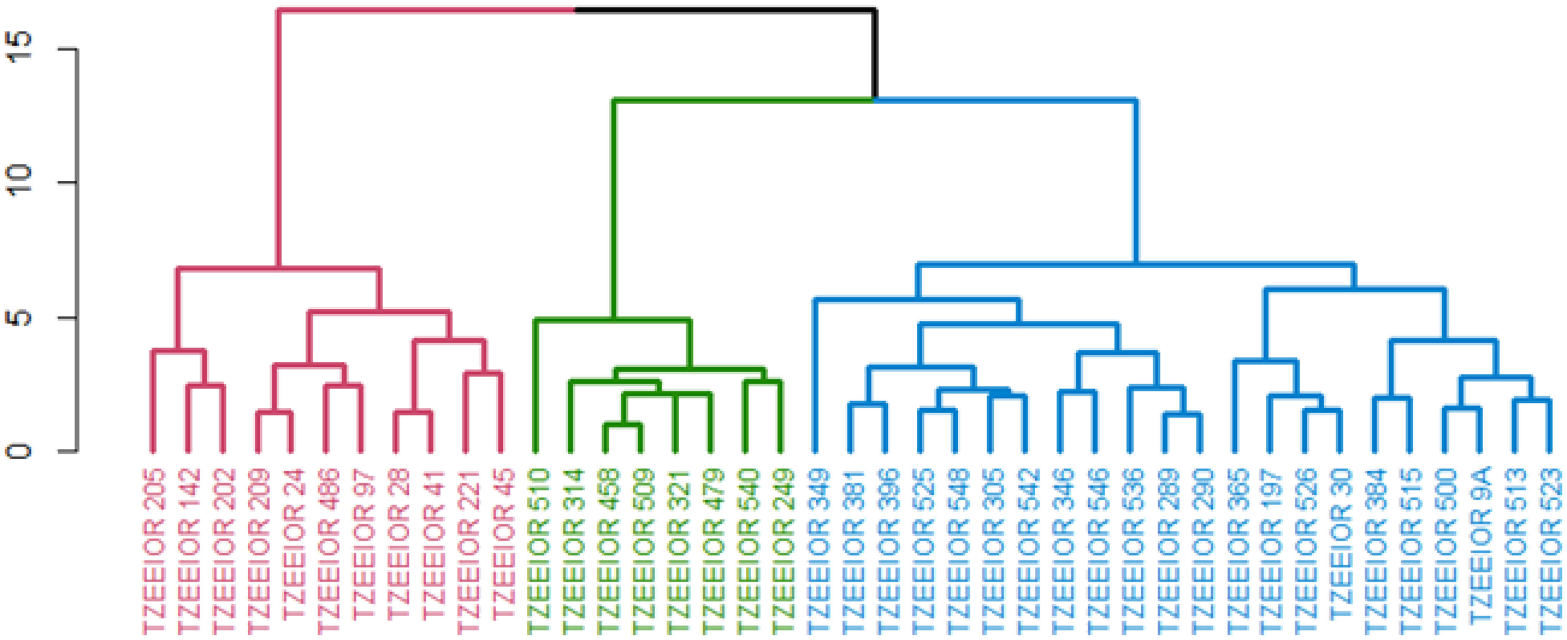
Heterotic group of 41 inbreds comprised 36 inbreds and 5 standard inbred testers under managed drought stress using the HGCAMT grouping method. Cluster 1 (Red), Cluster 2 (Blue), and Cluster 3 (Green).

Under managed drought conditions, the HGCAMT grouping method classified the 41 elite inbreds comprising 36 orange lines and 5 standard PVA testers into 3 heterotic groups. The first group had 11 inbred lines including the tester TZEEIOR 97. Except TZEEIOR 97 and TZEEIOR 205, the members of this group were characterized by negative GCA effects for GY, EPP, and STGC and positive GCA effects ASI, DP, DS, PASP, and EASP. The second cluster had the largest members (22 inbreds) comprising19 elite lines and 3 testers (TZEEIOR 197, TZEEIOR 9A, TZEEIOR 30). The members of this group were characterized by negative GCA effects for PHT and EHT. The third cluster consisted of eight inbreds, including tester TZEEIOR 249 is characterized by positive GCA effects for GY, EPP, and negative GCA effects for PASP, EASP, ASI, DP, and DS. Additionally, the majority of the inbreds in this group characterized by negative GCA effects for STGC except TZEEIOR 479 and TZEEIOR 540 (**Figure 2**).

**Figure 3:**
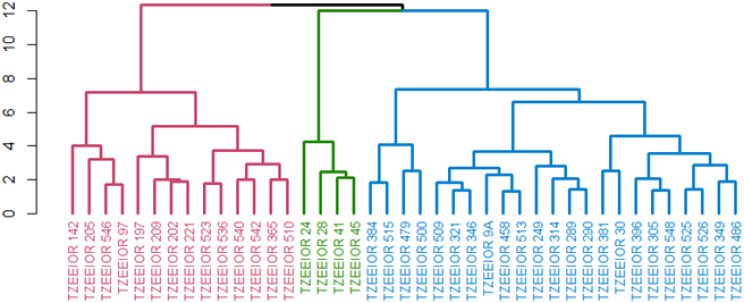
Heterotic group of 41 inbreds comprised 36 inbreds and 5 standard inbred testers under rain-fed conditions using the HGCAMT method. Cluster 1 (Red), Cluster 2 (Green), and Cluster 3 (Blue).

Under rain-fed conditions, three clusters were obtained. The first cluster comprised 14 inbred lines all characterized by positive GCA effects for GY with the exception of inbred TZEEIOR 209. The second cluster comprised four inbreds characterized by significant negative GCA effects for GY and EPP and positive GCA effects for ASI, DP, DS, PASP, EASP, and PHT. The third cluster consisted of 23 inbred lines including three testers (TZEEIOR 249, TZEEIOR 9A, and TZEEIOR 30). The members of this cluster had no features that were common to them (**Figure 3**).

**Figure 4:**
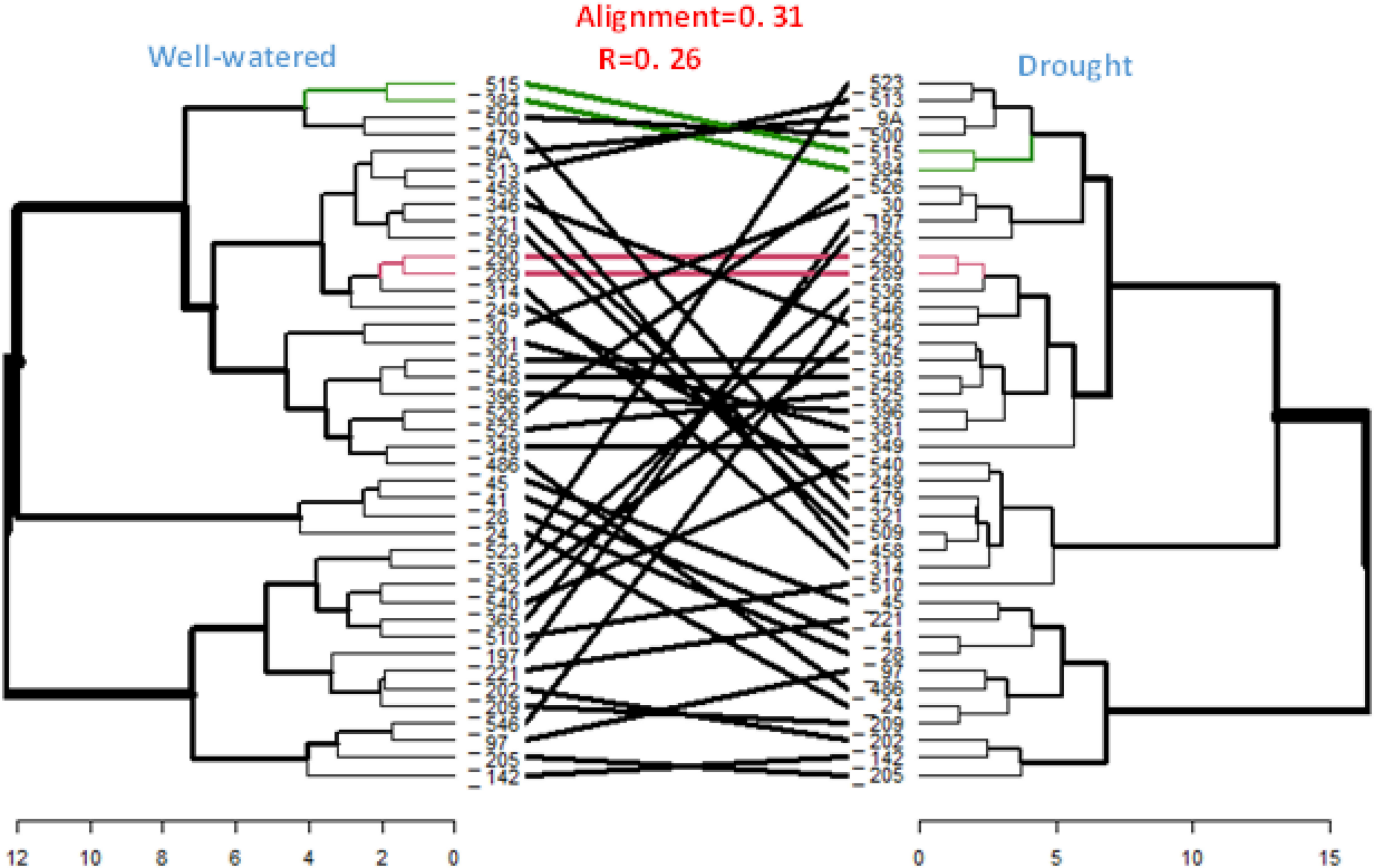
Comparison of heterotic groups of inbred lines using HGCAMT method under rain-fed and managed drought stress conditions. The black line in between the two dendrograms represent mismatched inbred lines while the red line are inbreds in the same position (perfect alignment) across both clusters and the green represent good a matched of inbred lines._: TZEEIOR; Well-watered=rain-fed.

Comparison between the hierarchical clusters derived from the GCA of the inbred lines under managed drought stress and rain-fed conditions showed a low cophenetic correlation (0.26) between the two hierarchical clusters (**Figure 4**). Only four out of the thirty-six inbred lines had the same position in both clusters.

### Performance of the testcross hybrids for grain yield and other agronomic traits under managed drought and rain-fed

Under managed drought conditions, the minimum GY was 198 kg/ha recorded by testcross TZEEIOR 41 × TZEEIOR 30 while the maximum GY was 4044 kg/ha obtained by TZEEIOR 509 × TZEEIOR 197 and the average GY 2139 kg/ha. TZEEIOR 509 × TZEEIOR 197, the best-yielding testcross showed 34% higher GY than TZEEI 79 × TZEEI 82, the best check which recorded a GY of 3015 kg/ha. ASI ranged from 0 to 4 days with a mean of 1.5 days. DP ranged from 45 days to 54 days and the mean was 50 days while DS varied between 45 days to 57 days with a mean of 51 days. EPP varied from 0.31 to 0.98 with a mean of 0.71. PASP varied from 3.83 to 7.39 with a mean of 5.42. EASP varied from 3.25 to 7 with a mean of 5.12. PHT ranged between 114.05.cm to 175 cm with an average of 147.86 cm. EHT ranged from 57.22 cm to 100.72 cm with an average of 76.36 cm (**Supplementary Table S5**).

Under rain-fed conditions, the minimum GY (1251 kg/ha) was observed for testcross TZEEIOR 28 × TZEEIOR 30 while the maximum (7455 kg/ha) was recorded by TZEEIOR 509 × TZEEIOR 197. The average GY was 5568 kg/ha. The best performing hybrid had grain yield increase of 30% over the best commercial check (5732 kg/ha) (**Supplementary Table S5**). ASI ranged from 0 to 2 days with an average of 0.61 days. DP ranged from 48 days to 54 days with an average of 51 days while DS varied between 47 days to 55 days with an average of 51 days. EPP varied from 0.44 to 1.07 with a mean of 0.92. PASP varied from 2.77 to 6.72 with a mean of 4.43. EASP varied from 3.25 to 6.25 with a mean of 4.78. PHT ranged between 147.88 cm to 209.47 cm with an average of 179.28 cm. EHT ranged from 64.75 cm to 110.05 cm with an average of 83.561 cm (**Supplementary Table S5**).

The grain yield loss in the testcross hybrids under managed drought stress compared with that of the rain-fed conditions varied from 35% (TZEEIOR 321 × TZEEIOR 249) to 89% (TZEEIOR 41 × TZEEEIOR 30) with a mean of 61% (**Supplementary Table S5**). Drought stress decreased PHT by 17%, EHT by 9%, EPP by 22%, DP by 2%, but extended ASI by 181%, DS by 0.2%. HCV, PASP, and EASP rating score increased by 7%, 25%, 11%, respectively (**Supplementary Table S5**).

### Performance of the parental inbred lines for grain yield and other agronomic traits under managed drought and rain-fed

Under managed drought conditions, GY of the inbred lines including the testers varied from 0 kg/ha for TZEEIOR 41 to 1409.5 kg/ha for TZEEIOR 540 with a mean of 577 kg/ha. ASI ranged from 0 days to 4.26 days with average of 1.5 days. DP ranged from 48.4 days to 60 days with an average of 54 days while DS varied between 47 days to 62 days with an average of 55 days. EPP varied from 0.07 to 0.97 with a mean of 0.55. PASP ranged from 4.02 to 7.33 with a mean of 5.47. EASP varied from 4.24 to 8.43 with a mean of 5.65. PHT ranged between 65.20 cm to 109.83 cm with an average of 85.47 cm. EHT ranged from 30.70 cm to 57.66 cm with an average of 41.67 cm (**Supplementary Table S6**).

Under rain-fed conditions, grain yield ranged from 703.8316 kg/ha for TZEEIOR 28 to 3757.34 kg/ha for TZEEIOR 513 with an average of 2025.91 kg/ha (**Supplementary Table S6**). ASI ranged from 0.24 days to 2.5 days with an average of 0.94 days. DP varied from 49.45 days to 60 days with an average of 54.47 days while DS varied between 48.47 days to 61 days with an average of 54.67 days. EPP varied from 0.55 to 1.31 with a mean of 0.88. PASP varied from 2.23 to 5.75 with a mean of 3.82. EASP varied from 2.75 to 6.31 with a mean of 4.26. PHT ranged between 92.07 cm to 146.86 cm with an average of 117.297 cm. EHT ranged from 34.14 cm to 64.44 cm with an average of 47.71 cm (**Supplementary Table S6**).

The grain yield penalty of the inbred lines under drought stress compared to that of rain-fed varied from 43% for TZEEIOR 515 to 100% for inbred TZEEIOR 41 with a mean of 71%. Drought stress contributed to decrease PHT by 27%, EHT by 12%, EPP by 37%, DP by 0.62% but prolonged ASI by 102%. PASP and EASP scoring rates increased by 48%, and 38%, respectively (**Supplementary Table S6**).

### Relationship between the mid-parent performance of parental inbreds and their corresponding hybrid

The correlation coefficient between mid-parent values and their corresponding hybrid means for GY and related traits under managed drought were significant (p<0.05) for all traits (diagonal values in **Table 3**). The absolute significant correlation coefficient varied from 0.26 for EHT to 0.72 for HCV, with 0.37 obtained for GY. Mid-parent values for DP, DS, ASI, PASP, and EASP had a significant (p<0.01) negative correlation with the GY of hybrids while the correlation coefficient between the mid-parent value for EPP and the grain yield of the hybrids was positive, and significant (0.38, **Table 3**).

**Table 3:** Correlation coefficient between mid-parental traits values of traits and their corresponding hybrids under managed drought stress.

Hybrid traits	Mid-parents values										
	GY	DP	DS	ASI	PHT	EHT	HCV	PASP	EASP	EPP	STGC
GY	0.37***	-0.31***	-0.4***	-0.36***	-0.06^ns^	0.05^ns^	0.18^ns^	-0.28***	-0.47***	0.38***	0.07^ns^
DP	-0.32***	0.6***	0.72***	0.4***	-0.24*	-0.24^ns^	-0.11^ns^	0.24*	0.31***	-0.46***	-0.33***
DS	-0.32***	0.54***	0.68***	0.42***	-0.04^ns^	-0.1^ns^	-0.28***	0.2*	0.36***	-0.42***	-0.33***
ASI	-0.32***	0.36***	0.47***	0.38***	0.19^ns^	0.09^ns^	-0.39***	0.11^ns^	0.41***	-0.3***	-0.27*
PHT	0.03^ns^	-0.12^ns^	-0.12^ns^	0.05^ns^	0.59***	0.46***	-0.44***	-0.25*	0.19^ns^	0.05^ns^	-0.08^ns^
EHT	-0.14^ns^	0.26*	0.27*	0.23*	0.13^ns^	0.26*	-0.16^ns^	-0.12^ns^	0.25*	-0.14^ns^	-0.41***
HCV	-0.33***	0.28***	0.1^ns^	0.01^ns^	-0.54***	-0.33***	0.72***	0.13^ns^	0.09^ns^	-0.25*	-0.17^ns^
PASP	-0.22*	0.32*	0.41***	0.28***	-0.23*	-0.27***	0.05^ns^	0.33***	0.24*	-0.32***	0.02^ns^
EASP	-0.35***	0.25*	0.33***	0.26*	0ns	-0.09^ns^	-0.1^ns^	0.30***	0.44***	-0.37***	-0.02^ns^
EPP	0.32***	-0.39***	-0.5***	-0.34***	0.23*	0.33***	0.03^ns^	-0.37***	-0.34***	0.48***	0.05^ns^
STGC	0.17^ns^	-0.21*	-0.17^ns^	-0.08^ns^	-0.14^ns^	-0.15^ns^	0.06^ns^	0.08^ns^	-0.12^ns^	0.17^ns^	0.31***

GY, grain yield; DP, days to 50% pollen shed; DS, days to 50% silking; ASI, anthesis-silking interval; PHT, plant height; EHT, ear height; HCV, husk cover; PASP, plant aspect; EASP, ear aspect testcross; EPP, ear per plant; STGC, stay green characteristic.

* = significant at 0.05 probability level; ***= significant at 0.001 probability level; ns= non-significant.

Under rain-fed conditions, the correlation coefficient between mid-parental values and their corresponding hybrid means for GY and other traits were significant (p<0.01) for GY, DP, DS, PHT, EHT, and HCV, but not significant for ASI, PASP, and EPP (Diagonal values in **Table 4**). In contrast to results obtained under managed drought, mid-parent values of PHT, EHT, and HCV had significant (p<0.01) and negative correlations with the GY of the hybrids. Similar to the results obtained under managed drought conditions, the mid-parent value for EPP was significant (p<0.01) and positively correlated to the GY of hybrids (r=0.34).

**Table 4:** Correlation coefficient between mid-parental values of traits and their corresponding hybrids under rain-fed condition.

Mid-parent values									
Hybrid trait	GY	DP	DS	ASI	PHT	EHT	HCV	PASP	EPP
GY	0.31***	-0.19^ns^	-0.16^ns^	0.12^ns^	-0.36***	-0.39***	-0.32***	-0.17^ns^	0.34***
DP	0.27***	0.58***	0.55***	-0.19ns	0.3***	0.14ns	-0.07ns	-0.01ns	0.12ns
DS	0.12^ns^	0.49***	0.59***	0.04^ns^	0.36***	0.14^ns^	-0.12^ns^	-0.02^ns^	-0.09^ns^
ASI	0^ns^	0.11^ns^	0.16^ns^	0.12^ns^	0.13^ns^	0.02^ns^	0^ns^	0.07^ns^	-0.04^ns^
PHT	0.11^ns^	0.33***	0.43***	0.14^ns^	0.51***	0.16^ns^	-0.17^ns^	-0.14^ns^	0.04^ns^
EHT	0.32***	0.32***	0.31***	-0.14ns	0.38***	0.36***	0.09ns	-0.11ns	0.2*
HCV	-0.07^ns^	0.24*	0.01^ns^	-0.58***	0.09^ns^	0.46***	0.63***	0.17^ns^	-0.05^ns^
PASP	-0.23*	0.08^ns^	0^ns^	-0.18^ns^	0.2^ns^	0.35***	0.38***	0.11^ns^	-0.16^ns^
EPP	0.15^ns^	-0.19^ns^	-0.11^ns^	0.32***	-0.1^ns^	-0.29***	-0.36***	-0.22*	0.18^ns^

GY, grain yield; DP, days to 50% pollen shed; DS, days to 50% silking; ASI, anthesis-silking interval; PHT, plant height; EHT, ear height; HCV, husk cover; PASP, plant aspect; EASP, ear aspect testcross; EPP, ear per plan.

* = significant at 0.05 probability level; ***= significant at 0.001 probability level; ns= non-significant.

Mid-parent heterosis and better-parent heterosis for GY were 338% and 247%, respectively, under managed drought stress and 173% and 137%, respectively under rain-fed conditions (**Table 5**). The grain yield of the F_1_ hybrids had a significant positive correlation with mid-parent value, better parent value, mid-parent heterosis, better-parent heterosis, and specific combining ability effects under managed drought stress. Under rain-fed conditions, the GY of the hybrids had a significant positive correlation with mid-parent, mid-parent heterosis, better-parent heterosis, and specific combining ability effects but the correlation with better-parent heterosis was not significant (**Table 6**).

**Table 5:** Mid-parent and better-parent heterosis for grain yield and other traits under managed drought and rain-fed conditions.

	Managed drought		Rain-fed	
	MPH	BPH	MPH	BPH
GY	337.9	246.6	172.9	136.8
DP	-10.0	-6.5	-7.8	-5.2
DS	-9.2	-5.1	-9	-5.3
ASI	5.6	201.2	-48.7	-57
PHT	66.1	53.1	46.1	34.4
EHT	81.6	67.8	67.8	53.4
HCV	23.8	44.9	20.4	38.4
PASP	3.6	13.2	23.0	41.4
EASP	-10.1	-1.8		
EPP	43.2	18.8	3.9	-4.2
STGC	-1.2	12.9		

GY, grain yield; DP, days to 50% pollen shed; DS, days to 50% silking; ASI, anthesis-silking interval; PHT, plant height; EHT, ear height; HCV, husk cover; PASP, plant aspect; EASP, ear aspect; EPP, ear per plant; STGC, stay green characteristic; MPH, mid-parent heterosis; BPH, better parent heterosis; MPH, mid-parent heterosis.

**Table 6:** Correlation coefficient among grain yield of F_1_, mid-parent value, better parent value, mid-parent heterosis, better-parent heterosis, and specific combining ability for hybrids derived from the 36 extra-early orange inbred lines under drought and rain-fed.

	Managed drought					Rain-fed				
	F_1_ GY	BP	MP	MPH	BPH	F_1_ GY	BP	MP	MPH	BPH
BP	0.36***					0.16ns				
MP	0.37***	0.91***				0.28***	0.86***			
MPH	0.23*	-0.66***	-0.71***			0.52***	-0.62***	-0.66***		
BPH	0.22*	-0.76***	-0.67***	0.89***		0.58***	-0.7***	-0.5***	0.89***	
SCA	0.67***	0.01^ns^	-0.03^ns^	0.45***	0.42***	0.57***	-0.04^ns^	0.04^ns^	0.41***	0.44***

F_1_ GY, grain yield of testcross; BP, better-parent; MP, mid-parent; MPH, mid-parent heterosis; BPH, better-parent heterosis; SCA, specific combining ability.

* = significant at 0.05 probability level; ***= significant at 0.001 probability level; ns= non-significant.

Under managed drought stress, 16 testcross hybrids out-yielded the best commercial check. The least heterosis based on the yield of the best commercial check was observed in the hybrid cross TZEEIOR 523 × TZEEIOR 97 (1.3%) while the highest was obtained in the hybrid cross TZEEIOR 509 × TZEEIOR 197 (34.1%) (**Table 7**).

**Table 7:** Best testcrosses under drought stress compared to the best commercial check.

ntries	TC	Grain yield of TC (kg/ha)	ECH (%)
121	TZEEIOR 509 × TZEEIOR 197	4044.2	34.2
179	TZEEIOR 510 × TZEEIOR 30	3805.5	26.3
118	TZEEIOR 458 × TZEEIOR 197	3643	20.9
39	TZEEIOR 314 × TZEEIOR 97	3604.2	19.6
70	TZEEIOR 510 × TZEEIOR 97	3569	18.4
175	TZEEIOR 536 × TZEEIOR 30	3498.1	16.1
149	TZEEIOR 384 ×TZEEIOR 30	3415	13.3
68	TZEEIOR 526 × TZEEIOR 97	3318.8	10.1
106	TZEEIOR 510 × TZEEIOR 249	3294.8	9.3
159	TZEEIOR 540 × TZEEIOR 30	3261.2	8.2
178	TZEEIOR 525 × TZEEIOR 30	3217.1	6.7
74	TZEEIOR 321 × TZEEIOR 249	3207.9	6.4
110	TZEEIOR 321 × TZEEIOR 197	3116.3	3.4
158	TZEEIOR 509 × TZEEIOR 30	3097	2.8
119	TZEEIOR 479 × TZEEIOR 197	3073.8	2
52	TZEEIOR 523 × TZEEIOR 97	3054.1	1.3

TC, testcross; BCC, best commercial check; ECH (%), economic heterosis; Grain yield of best commercial check is 3015.2 kg/ha.

## Discussion

This study provides an insight into the inheritance of tolerance to drought in newly developed orange kernel Provitamin A extra early maize inbred lines. In addition to the identification of drought-tolerant hybrids, made possible by the evaluation of a large number of testcrosses and their parental inbred lines, the results obtained have the potential for guiding the development of hybrids that are superior to those identified in the present study under both drought and rain-fed growing conditions.

Large genetic differences for grain yield and important agronomic traits among the 180 extra-early orange testcross hybrids were supported by the significant mean squares obtained for the studied traits under managed drought stress and rain-fed conditions. These results offer the opportunity for the selection of testcross hybrids with the desirable combination of traits under each of the growing conditions studied. Similar to the results obtained in this study, Oluwaseun et al. (2022) also reported significant genetic variation among 132 extra-early maturing provitamin A maize hybrids. As expected, the results obtained were different for the two seasons in which the studies were carried out under each managed drought and non-drought conditions. In addition to moisture, which directly affects relative humidity, other environmental factors such as temperature, photoperiod, and solar radiation exhibit significant seasonal variation in tropical regions. These factors, and their interactions, create distinct environmental conditions for each season (Adetimirin, 2024).

In this study, we analyzed genotype-by-environment (GEI) interactions for eleven traits under two growing conditions. The overall GEI effect was non-significant for many traits. However, a closer examination of the individual components (GCA_Line × environment, GCA_Tester × environment, and SCA Line × Tester × environment) revealed significant interactions for seven traits under drought conditions and eight traits under rain-fed conditions. Under drought, this was evidenced by significant effects for 4 and 8 out of 11 traits for GCA Line × environment interaction and GCA Tester × environment interaction, respectively. Under rain-fed conditions, significant effects were obtained for 4 out of 10 traits for GCA_Line × environment interaction and 6 out of 10 traits for GCA Tester × environment interaction. In effect, the lines and testers differed in their response patterns to the environmental conditions for many of the traits. Similar to this results, significant GEI for grain yield, plant and ear aspects, and plant and ear height in drought and optimum research conditions were reported for 96 early provitamin A quality protein hybrids and 252 extra-early white single cross maize hybrids (Obeng-Bio et al., 2019; Amegbor et al., 2020). Genotype × environment interaction is heritable, and genotypes may show differences for this component of their genetic make-up. It is imperative, therefore, to conduct agronomic evaluation in at least two seasons as the results. In addition to providing important information on the pattern of response to changes in environmental conditions, it also provides information on the magnitude of variation in effect that the environment is capable of exerting.

One of the advantages of the mating design and analysis used in this study is the possibility of decomposing testcross performance into GCA for the lines and testers, and SCA for their crosses. This is important as it separates the performance of parental lines into effects that could be transmitted to progenies from those that are derived from the unique combination of the lines in crosses. The significance of GCA for lines, testers, and SCA for almost all measured traits under managed drought and rain-fed conditions are indicative of genetic variation among the lines and testers, and the importance of additive and non-additive genetic variances. For these traits, a greater importance for additive compared to non-additive genetic variances was, however, indicated by the higher percentage of the mean square accounted for by GCA variance. Results similar to these were reported by other authors who studied extra-early and intermediate maize of West African adaptation and maize hybrids adapted to Southern Africa under drought, combined drought and heat stress, and rain-fed conditions (Derera et al., 2008; Oluwaseun et al., 2022; Mboup et al., 2023).

Our results and previous research on the importance of additive over non-additive variance clearly indicate that S_1_ family and full-sib family selection schemes are effective for improving grain yield of maize for drought tolerance. These results also indicate that the potential of maize lines could be reliably evaluated during early generation testing of parental lines, given the preponderance of GCA over SCA variances. However, the significant SCA variances indicate that the ultimate potential of each line could only be determined in hybrid combinations at advanced stages of inbreeding when the lines have attained homozygosity. The decomposition of the genetic variances also provides an understanding of the components of genetic variation that interacted more with environment. The results of this study showed that the GCA component of genotypic variance interacted much more with the environment than the SCA component, with a higher interaction obtained for the tester with environment than interaction of the line with the environment. In the present study, the SCA x environment interaction was indicated by Line x Tester x environment interaction for which significance was obtained for only the ear aspect under managed drought while non-significance was observed for all traits under rain-fed conditions.

Many studies have identified components of grain yield under stress (biotic and abiotic) and non-stress conditions as EPP, EASP, and PASP (Adetimirin et al., 2000). In addition to these traits, ASI and STGC are important indicators of tolerance to moisture stress and other abiotic stresses and are considered important selection indices under these conditions (Maazou et al., 2016). Low GCA value, whether positive or negative, for an inbred is indicative of little variation between the means of the parents in the cross combinations and the overall mean of the crosses. A high GCA value, reflects a considerable difference between the mean performance of a parent crossed to other parents and the overall mean (Ahmed and Mahmoud, 2015; Fasahat et al., 2016; Quirino et al., 2020). Under moisture stress, desirable inbreds are those with significant and positive GCA effects for GY and EPP, and significant negative GCA effects for EASP, PASP, and STGC. Such inbreds are capable of transmitting their desirable characteristics to their offspring. In the present study, only inbred TZEEIOR 510 showed all these attributes, indicating the inbred as having a perfect combination of genes for desirable traits and valuable in the breeding for tolerance to drought. Inbred TZEEIOR 28 had significant and negative GCA effects for the stay-green characteristic, an indicator of delayed senescence under managed drought. This inbred could be invaluable for use in the introgression of genes for the desirable stay-green characteristics in the extra-early orange maize populations. Since the occurrence of drought is unpredictable, with moisture stress occurring in some seasons and absent in others, genotypes suitable for selection must possess desirable and significant GCA effects for grain yield and the yield components under non-stress combinations.

It is noteworthy that inbreds TZEEIOR 28, TZEEIOR 41, TZEEIOR 45, and TZEEIOR 24, which had significant negative GCA effects for grain yield under rain-fed conditions in the present study, were also reported in another study to have very poor grain yield under both managed drought and optimum conditions (Bonkoungou et al., 2024). Inbred TZEEIOR 24 was identified as drought-tolerant in a previous study (Badu-Apraku et al., 2020), but its tolerance to drought was not apparent in the present study. Tolerance to drought is a complex trait, and tolerance response to managed drought may vary depending on the severity and duration of the latter from season to season due to uncontrolled confounding effects of other environmental factors such as heat. None of the testers used in this study showed significant GCA effects for grain yield and other traits under managed drought and rain-fed conditions. These results are indicative of similar productivity of the testers under the conditions studied.

The estimates of broad-sense heritability obtained for most of the traits were moderate to high. These results demonstrate the effective reduction of environmental variation in our study. The variation obtained were due largely to genetic differences, and indicated the likelihood of selection success based on phenotypic performance for the genotypes evaluated (Aminu and Izge, 2012). The known yield components under managed drought stress in the present study, namely EPP, EASP, and STGC, had estimates of narrow sense heritability comparable to that obtained for GY. These results indicate that in situations where environmental variation is successfully controlled as in the present study, indirect selection for yield components may not have any advantage over direct selection for grain yield. However, these traits have value for the development of a base index for the identification of drought tolerance and have been so used in previous studies (Ziyomo and Bernardo, 2013; Badu-Apraku et al., 2015).

The inbred lines clustered into three groups under each of both managed drought and rain-fed conditions using the HGCMT method. Heterotic grouping is a strategy for optimizing the hybrid development process, obviating the need to test developed inbred lines in all possible combinations. In effect, crosses are not made among inbred lines within each group but between inbred lines in different heterotic groups. In addition to establishing heterotic groups, in the present study inbred lines with less desirable features were clustered into the same heterotic group. These inbred lines are those in cluster one under managed drought and cluster two under rain-fed conditions. The low correlation between the dendrogram obtained under drought and rain-fed conditions indicate that heterotic grouping under the two conditions studied were not similar.

In the present study, we imposed drought towards the flowering and continued throughout the grain-filling stages. This negatively affected various plant characteristics, ultimately reducing grain yield. The stress prolonged the anthesis-silking interval, decreased the number of ears per plant, and affected all the visual scoring traits (PASP, EASP). These factors collectively contributed to reduce the grain production. According to Lima et al. (2023), a prolonged anthesis-silking interval increases the incidence of barrenness, contributing to yield loss. The yield losses observed in this study ranged from 35% to 89%, averaging 61% for the hybrids and 43% to 100% with an average of 71% for the inbred lines. The high yield reduction observed is due to the duration of drought imposed. These results align with the findings from previous research (Bonea, 2020; Lima et al., 2023).

The importance of parental performance, indicated by the significant and positive correlation between mid-parental values and hybrid mean performance indicated that hybrid performance could be reliably predicted from the performance of the inbred parents. Predicting hybrid performance based on the performance of their parental lines is important for significant gains from selection (Oyekunle, 2014; Chung and Liao, 2022). The significant and positive correlation of the grain yield of the F_1_ with heterosis, especially better-parent heterosis, and specific combining ability underscores the importance of heterosis and specific combining ability in the performance of the testcrosses. According to Betrán et al (2003), the grain yield of a hybrid can be predicted using both heterosis and combining ability. The mid-parent and better-parent heterosis observed in the present study are much higher under managed drought than under rain-fed conditions. In effect, the superiority of the inbred lines was more evident under drought stress. Environmental factors are known to influence heterosis to different degrees in both inbred parents and hybrids (Betrán et al., 2003). The 16 testcrosses that outyield the best commercial hybrid check are promising for further evaluation and commercialization to address drought and vitamin A deficiency in the West and Central Africa sub-region.

## Conclusion

The genetic studies presented in this paper were based on the orange kernel inbred lines selected from the pool of inbreds improved for PVA content at the IITA maize-breeding program. The study revealed that under managed drought, TZEEIOR 510 showed positive and desirable (positive) GCA effects for grain yield and ears per plant, and significant and desirable (negative) GCA effects for plant, ear aspects and stay green characteristic. Both additive and non-additive gene actions were responsible for the inheritance of grain yield and related traits under managed drought and rain-fed conditions in extra-early orange inbreds. However, there is a preponderance of additive gene action over non-additive gene action. Significant and positive correlation coefficients were obtained between grain yield of testcross hybrids and heterosis on the one hand, and specific combining ability on the other hand, indicating that superior testcross performance was a function of heterosis. Three heterotic groups were defined for the inbreds lines used for the study under each of the growing conditions based on the HGCAMT procedure. Sixteen testcross hybrids had higher grain yield than the best commercial check. Of this 16, eight testcrosses had more than 10% yield advantage over the best check. The inbred lines used in this study showed good potential for carotenoid hybrid development to mitigate the increasing chronic food and nutritional inadequacies in West and Central Africa.

## Data Availability

The datasets presented in this study can be found in online repositories. The names of the repository/repositories and accession number(s) can be found in the article/**Supplementary Material.**
